# MicroRNAs in microglia: deciphering their role in neurodegenerative diseases

**DOI:** 10.3389/fncel.2024.1391537

**Published:** 2024-05-15

**Authors:** Shweta Pradip Jadhav

**Affiliations:** Thermo Fisher Scientific, Waltham, MA, United States

**Keywords:** miRNA, microglia, neuroinflammation, neurodegeneration, AD, PD, ALS

## Abstract

This review presents a comprehensive analysis of the role of microRNAs in microglia and their implications in the pathogenesis of neurodegenerative diseases. Microglia, as the resident immune cells of the central nervous system (CNS), are pivotal in maintaining neural homeostasis and responding to pathological changes. Recent studies have highlighted the significance of miRNAs, small non-coding RNA molecules, in regulating microglial functions. In neurodegenerative diseases, such as Alzheimer’s Disease (AD), Parkinson’s Disease (PD), Amyotrophic Lateral Sclerosis (ALS), and Multiple Sclerosis (MS), dysregulated miRNA expression in microglia contributes to disease progression through various mechanisms such regulation of gene expression, as modulation of cytokine response and phagocytosis. This review synthesizes current knowledge on how miRNAs influence microglial activation, cytokine production, and phagocytic activity. Specific miRNAs, such as miR-155, are explored for their roles in modulating microglial responses in the context of neuroinflammation and neurodegeneration. The study also discusses the impact of miRNA dysregulation on the transition of microglia from a neuroprotective to a neurotoxic phenotype, a critical aspect in the progression of neurodegenerative diseases.

## 1 Introduction

Neuroinflammation is a pivotal process in the CNS, encompassing a wide range of mechanisms and responses triggered by various neurological disorders, injuries, and infections. This complex biological response involves immune cells, glial cells, cytokines, and chemokines, aiming to remove harmful stimuli and initiate tissue repair. However, when dysregulated, neuroinflammation can contribute to the progression of various neurodegenerative diseases, including Alzheimer’s disease, Parkinson’s disease, multiple sclerosis (MS), and amyotrophic lateral sclerosis (ALS) (Aktas et al., 2007; Aïd and Bosetti, 2011).

At the heart of neuroinflammation are glial cells, primarily microglia and astrocytes, which are the resident immune cells of the CNS. In a healthy brain, microglia act as sentinels, constantly surveying their environment for signs of disturbance. Upon detecting injury or disease, these cells quickly become activated. They can release cytokines and chemokines, which are signaling molecules that can promote or inhibit inflammation. Additionally, they can phagocytose, or engulf, cellular debris and pathogens, aiding in clearing the brain of detrimental substances (Butovsky and Weiner, 2018).

The role of neuroinflammation is multifaceted and can be both beneficial and detrimental. On the one hand, it is crucial for the CNS’s defense against infections and for repairing tissue damage. In the acute phase, neuroinflammation helps to isolate and remove pathogens or damaged cells and begins the process of tissue repair. On the other hand, chronic neuroinflammation is a common pathological feature in many neurodegenerative diseases. Prolonged inflammation can lead to the sustained release of pro-inflammatory cytokines and other harmful substances, which can contribute to neuronal damage and loss (Niranjan, 2018; Guo et al., 2022).

In Alzheimer’s disease, neuroinflammation is evident by the presence of activated microglia and astrocytes surrounding amyloid plaques (Gerrits et al., 2021). Similarly, in Parkinson’s disease, neuroinflammatory processes are associated with degeneration of dopaminergic neurons (Ho, 2019). In MS, an autoimmune disorder, neuroinflammation is characterized by the infiltration of immune cells from the bloodstream into the CNS, damaging the myelin sheath that insulates nerve fibers (Yong, 2022). In ALS, it is linked with the progression of motor neuron damage (Geloso et al., 2017).

Recent research in neuroinflammation has focused on understanding the delicate balance between its protective and harmful effects. This includes studying the molecular and cellular mechanisms underlying the initiation and resolution of neuroinflammation, as well as identifying how these processes go awry in neurodegenerative diseases. There is also a growing interest in developing therapies that can modulate neuroinflammatory responses, aiming to harness the beneficial aspects of inflammation while minimizing its detrimental effects. Understanding microglial-mediated neuroinflammation and its impact on CNS health and disease continues to be a critical area of research, holding the promise for innovative therapeutic approaches to a range of neurological disorders.

## 2 Role of microglia in the CNS

Microglia are highly dynamic cells, constantly surveying their environment for signs of disturbance or damage. In the healthy brain, they contribute to homeostasis by clearing dead cells and debris, regulating synaptic pruning, and supporting neuronal survival. Microglia also play a crucial role in shaping brain development and modulating synaptic connectivity (Colonna and Butovsky, 2017). Upon detecting signals of injury, infection, or disease, microglia rapidly transition from a “resting” state to an “activated” state. This activation is characterized by changes in their morphology (from ramified to amoeboid shape), upregulation of surface receptors, and secretion of various molecules, including cytokines, chemokines, and neurotrophic factors. Activated microglia can exhibit a spectrum of functional states, ranging from pro-inflammatory (often termed M1) to anti-inflammatory (often termed M2), although this classification is overly simplistic given the diversity of microglial phenotypes observed in different contexts (Li and Barres, 2018).

Microglial activation is a key component of the neuroinflammatory response. In the acute phase, this response is typically protective. Microglia remove cellular debris, secrete factors that promote tissue repair, and orchestrate the recruitment of other immune cells if necessary. However, if the activation of microglia is sustained or dysregulated, it can contribute to chronic neuroinflammation, which is associated with various neurodegenerative diseases (Subhramanyam et al., 2019).

In chronic neuroinflammation, microglia can release excessive amounts of pro-inflammatory cytokines (TNF-α, IL-1β, and IL-6) and reactive oxygen species (ROS). This prolonged inflammatory response can lead to neuronal damage and exacerbate disease progression (Subhramanyam et al., 2019). Given their central role in neuroinflammation, microglia have become a target for therapeutic intervention in neurodegenerative diseases. Therapies aimed at modulating microglial activation, promoting their neuroprotective functions, or inhibiting their pro-inflammatory actions are under investigation. The challenge lies in selectively targeting harmful aspects of microglial activation without compromising their beneficial roles in CNS homeostasis and repair.

## 3 microRNA biology

MicroRNAs (miRNAs) are short, regulatory RNA molecules that play a key role in controlling gene expression after transcription. They are produced from primary miRNAs (pri-miRNAs) in the nucleus, which are processed into precursor miRNAs (pre-miRNAs) and then into mature miRNAs in the cytoplasm. Mature miRNAs guide the RNA-induced silencing complex (RISC) to target mRNAs, leading to repression or degradation of these mRNAs. This process allows miRNAs to regulate various cellular functions, including growth, differentiation, and apoptosis. Due to their ability to target multiple genes and their involvement in numerous diseases, miRNAs are considered important for maintaining cellular balance and have emerged as potential therapeutic targets for various conditions (Kim and Nam, 2006).

### 3.1 microRNAs in microglia

MiRNAs in microglia represent an emerging and significant area of study in neurobiology, offering insights into how these small non-coding RNA molecules influence the functioning and behavior of these crucial immune cells in the CNS. Microglia, as the resident macrophages of the brain, play pivotal roles in maintaining CNS homeostasis, responding to injury and disease, and modulating neuroinflammation. The regulatory functions of miRNAs in microglia are integral to these processes, impacting their activation, proliferation, and response to pathological stimuli (Karthikeyan et al., 2016).

### 3.2 Role of miRNAs in microglial function

- Regulation of Microglial Activation: miRNAs can modulate the activation state of microglia. Certain miRNAs are known to promote the classical (pro-inflammatory) or alternative (anti-inflammatory) activation states by targeting specific signaling pathways. For instance, specific miRNAs can downregulate the expression of pro-inflammatory cytokines, thereby suppressing the inflammatory response, which is crucial in the context of neuroinflammatory diseases (Guedes et al., 2013).
- Involvement in Neuroinflammation: In neurodegenerative diseases like Alzheimer’s disease (AD) and Parkinson’s disease (PD), miRNAs in microglia have been shown to influence the disease pathology. Dysregulation of specific miRNAs in microglia can contribute to neuroinflammatory processes, exacerbating neuronal damage. For example, certain miRNAs might modulate the microglial response to amyloid-beta in AD, affecting plaque clearance and inflammatory responses (Li et al., 2023).
- Impact on CNS Homeostasis: Microglial miRNAs are also involved in normal brain development and maintenance. They can influence microglial surveillance functions, synaptic pruning, and interactions with neurons, thereby contributing to the fine-tuning of neural circuits and supporting cognitive functions (Michell-Robinson et al., 2015).
- Response to CNS Injury: Following injury, miRNAs in microglia can modulate the cells’ response to damage. This includes shifts in microglial activation states and the release of factors that can either promote repair and regeneration or contribute to secondary damage (Wei et al., 2016).

## 4 Specific microRNAs modulating microglial function in neurodegenerative disorders

### 4.1 AD

Abnormal microglial activation is a hallmark of Alzheimer’s Disease (AD) progression, with microglia shifting from a neuroprotective to a pro-inflammatory phenotype under AD conditions, exacerbating neuronal damage and disease pathology. Research into miRNAs has illuminated their role in this process and their potential as therapeutic targets:

- Li et al. (2011) found that miRNA-146a inversely affects CFH expression in human microglial cells, indicating a potential mechanism for uncontrolled inflammation in AD.
- Liang et al. (2021) demonstrated that miR-146a overexpression in microglia benefits APP/PS1 transgenic mice by reducing cognitive impairments and neuroinflammation, suggesting miR-146a’s therapeutic potential.
- Wan et al. (2021) observed that enhancing miR-191-5p improves microglial survival and reduces apoptosis in AD models by targeting Map3k12, indicating a protective mechanism against microglial damage.
- Xing et al. (2016) identified the miR-206/IGF1 signaling pathway as a contributor to microglial inflammation in AD, offering a new target for therapy.
- Alexandrov et al. (2013) reported that aluminum exposure increases miRNA-34a levels, reducing TREM2 expression via NF-kB activation, with implications for AD pathology. This effect can be counteracted by CAPE, highlighting a novel epigenetic pathway in AD.
- Walgrave et al. (2023) found that miR-132 restoration in AD models improves amyloid and Tau pathologies, suggesting its role in transitioning microglia to a homeostatic state and its therapeutic promise. Research by Aloi et al. (2021, 2023) and others on miR-155 underscores its complex role in AD, affecting β-Amyloid processing, microglial activation states, and neural network stability, with implications for therapeutic targeting to balance neuroinflammation and cognitive functions.

### 4.2 ALS

Recent research has significantly advanced our understanding of the role of miRNAs in ALS, particularly focusing on miR-125b and miR-155. Parisi et al.’s (2016) study highlighted how miR-125b in microglia affects NF-κB signaling in ALS, uncovering a detrimental mechanism where miR-125b suppression leads to overactive NF-κB and consequent motor neuron damage. They found that inhibiting miR-125b can improve motor neuron survival, suggesting a new therapeutic approach for ALS (Parisi et al., 2016).

Complementing this, Butovsky et al. (2015) explored the impact of miR-155 in SOD1 mice, a standard ALS model. They observed increased miR-155 expression linked to impaired microglial functions and discovered that deletion of miR-155 not only extended the lifespan of these mice but also normalized their microglial profiles. Additionally, they found that miR-155 levels were elevated in familial and sporadic ALS patients, and targeting miR-155 therapeutically showed promise in prolonging survival (Butovsky et al., 2015).

Together, these studies illuminate the crucial roles of miR-125b and miR-155 in modulating microglial activity in ALS. They open potential avenues for miRNA-based treatments aimed at mitigating neuroinflammation and protecting motor neurons in ALS, marking significant progress in the quest for effective ALS therapies.

### 4.3 PD

Parkinson’s Disease (PD) is characterized by the decline of dopamine-producing neurons and the accumulation of Lewy bodies, leading to motor dysfunction (Emamzadeh and Surguchov, 2018). A key factor in PD’s progression is the activation of microglia and subsequent neuroinflammation, particularly through the NLRP3 inflammasome pathway in the substantia nigra.

Research has shed light on various microRNAs (miRNAs) offering potential therapeutic targets for PD by modulating neuroinflammation and microglial activation:

- Sun et al. (2019), found that miR-190, which targets Nlrp3, is downregulated in PD models but its overexpression can reduce inflammation and protect dopaminergic neurons.
- Yao et al. (2018) demonstrated that miR-124 can mitigate neuroinflammation and neuronal apoptosis by influencing the MEKK3/NF-κB pathways, highlighting its therapeutic potential.
- Zhou et al. (2016) identified miR-let-7a’s role in reducing microglial activation and improving motor and cognitive functions in PD mice by targeting STAT3, suggesting miR-let-7a as a crucial regulator of neuroinflammation.
- Gong et al. (2022) observed that miR-132-3p overexpression enhances microglial activation, a process that can be reversed by GLRX overexpression, indicating the regulatory relationship between miR-132-3p and GLRX in PD.
- Wang et al. (2024), found that miR-218-5p overexpression can counteract microglial inflammation and the upregulation of type I interferon pathways, with Ddx41 identified as a direct target.
- Wang et al. (2020) showed that enhancing miR-29c levels can decrease LPS-induced pro-inflammatory responses, with NFAT5 acting inversely to miR-29c, underscoring miR-29c’s potential for PD treatment.

These studies collectively highlight the significant impact of miRNAs in regulating neuroinflammation and microglial activation in PD, offering promising directions for developing new therapeutic strategies.

### 4.4 MS

Recent research by Fang X et al., Li Y et al., and Yu Z et al. has underscored the critical roles of miRNAs in MS through their regulation of microglial behavior in the central nervous system. Fang X et al. found that miR-30a exacerbates MS by promoting pro-inflammatory responses, suggesting it as a target for reducing microglial-induced inflammation (Fang et al., 2017). Li Y et al. discovered that the absence of miR-223 lessens MS symptoms by enhancing autophagy, with miR-223 targeting ATG16L1, indicating its potential as a therapeutic target (Li et al., 2019). Yu Z et al. showed that miR-126a-5p maintains blood–brain barrier integrity and that its deletion worsens MS, but its protective effects can be mimicked with MMP9 inhibition or Auranofin treatment, pointing toward miR-126a-5p modulation as a therapeutic strategy (Yu et al., 2022). Collectively, these findings highlight the importance of miRNAs in MS pathogenesis and therapy, offering new avenues for treatment through microglial modulation.

## 5 Conclusion

In conclusion, the intricate interplay between miRNAs and microglial activation within the context of neuroinflammation presents a promising frontier in the quest to understand and treat neurodegenerative diseases. The emerging research on miRNAs offers valuable insights into the molecular mechanisms driving microglial behavior, revealing potential targets for modulating neuroinflammatory responses in disorders such as AD, PD, ALS, and MS. Profiling miRNAs in body fluids like blood and CSF could revolutionize the diagnosis and prognosis of neurodegenerative diseases by providing accessible, non-invasive biomarkers. The development of miRNA-based therapies, including combinatory gene therapy, represents a significant step towards personalized medicine, offering new avenues for treating neurological disorders.

The studies reviewed here highlight the significant impact of specific miRNAs, such as miR-146a, miR-155, miR-190, miR-124, and miR-30a, on the regulation of microglial functions—from influencing the cells’ activation states to modulating their responses to pathological stimuli. By either promoting or inhibiting neuroinflammatory processes, these miRNAs directly affect disease progression and neuronal survival, offering new avenues for therapeutic intervention.

Future research aimed at further elucidating the roles of miRNAs in neuroinflammation and microglial activation is essential. Such studies could pave the way for developing innovative miRNA-based therapies that harness the protective aspects of neuroinflammation while mitigating its harmful effects. Ultimately, understanding the nuanced roles of miRNAs in neurodegenerative diseases holds the promise of uncovering novel therapeutic strategies that could significantly alter the course of these debilitating conditions, improving outcomes for patients worldwide.

## Author contributions

SPJ: Conceptualization, Data curation, Methodology, Resources, Writing – original draft, Writing – review & editing.
